# Does Disclosure About the Common Factors Affect Laypersons' Opinions About How Cognitive Behavioral Psychotherapy Works?

**DOI:** 10.3389/fpsyg.2018.02635

**Published:** 2018-12-21

**Authors:** Charlotte R. Blease, John M. Kelley

**Affiliations:** ^1^General Medicine and Primary Care, Beth Israel Deaconess Medical Center, Harvard Medical School, Harvard University, Boston, MA, United States; ^2^School of Psychology, University College Dublin, Dublin, Ireland; ^3^School of Psychology, Endicott College, Beverly, MA, United States

**Keywords:** common factors, psychotherapy, informed consent, cognitive behavioral therapy, clinical Ethics, informational privacy, confidentiality in psychotherapy, ethics Education and training

## Abstract

**Background:** Written and online information about cognitive-behavioral therapy (CBT) prioritizes the role of specific techniques (e.g., cognitive restructuring) and typically omits discussion of “common factors” (e.g., the working alliance, or therapist empathy). However, according to extensive psychotherapy process research the common factors may be important mediators of client improvement.

**Objectives:** This study aimed to assess lay opinions about the role of specific and common factors in CBT for depression. We also aimed to determine how different client disclosure processes might affect lay opinions about the relative importance of specific and common factors in CBT.

**Methods:** We conducted a web-based experiment involving a sample of US participants who had never undergone psychotherapy. All participants were presented with similar vignettes describing an individual suffering from depression whose doctor recommends CBT. Participants were randomized to read one of six vignettes created in a 2 × 3 factorial design that crossed client gender with type of informed consent (Standard CBT Disclosure vs. Common Factors and CBT Disclosure vs. No Disclosure).

**Results:** Disclosure type had a significant effect on participants' ratings of Common and Specific factors in psychotherapy. As compared to the CBT disclosure, participants allocated to the Common Factors disclosure rated Empathy and Positive Regard as significantly more important to treatment outcome, and rated the Specific factors of CBT as significantly less important to outcome. There were no significant differences between No Disclosure and Standard CBT Disclosure, and these participants rated Specific factors of CBT and the Working Alliance as more important components in treatment, and Empathy and Positive Regard as less important.

**Conclusions:** The content of information disclosures influences lay opinions about the importance of specific and common factors in CBT. Further research should investigate ethically acceptable disclosures to CBT and other forms of psychotherapy, including whether disclosure practices affect treatment outcome.

## Introduction

Cognitive behavioral psychotherapy (CBT) is the fastest growing and most widely used form of psychotherapy worldwide, practiced by a quarter of all therapists (Norcross et al., 2005). In 2007, the UK government invested £300 million with its Improved Access to Psychological Treatments' (IAPT) plan. The IAPT plan aimed to train 6,000 CBT therapists to improve access to psychotherapy for National Health Service clients in England (Clark, 2011). The effectiveness of CBT for depression is well-established, although there is ongoing discussion about whether CBT outperforms other versions of psychotherapy in the treatment of depression (Smith and Glass, 1977; Leichsenring et al., 2004; Fonagy et al., 2015). Meta-analyses show that around 80% of people who undergo any version of psychotherapy are better off than clients who receive no treatment (Cuijpers et al., 2008; Wampold and Imel, 2015). These effect sizes are comparable to antidepressants, but there is evidence that CBT for depression is a more enduring treatment than pharmacological interventions because it prevents relapse (Hollon et al., 2006). This paper focuses on a less investigated issue in clinical psychology and psychotherapy: the content of information provided to individuals about CBT during informal and formal information disclosure (Gaab et al., 2016; Trachsel and Gaab, 2016; Blease et al., 2018). In particular, we examine whether the content of written information provided to lay participants influences lay opinions about the effectiveness of CBT and how it works.

### Informed Consent to Psychotherapy

Historically, there has been limited ethical (Johnson-Greene, 2007; Blease et al., 2016c) and empirical analysis of the process and content of informed consent in psychotherapy, though this has begun to change in recent years. Ethics codes of professional organizations such as the American Psychological Association (2010), American Medical Association (2006), the General Medical Council (2010) (in the UK), and the British Association for Counseling and Psychotherapy (2016) state that practitioners and therapists are duty bound to provide adequate informed consent to clients.

However, there is considerable latitude for interpretation, both in respect to what is conveyed to prospective clients and how consent is obtained. A limited number of qualitative and quantitative studies in the US and UK reveal variation among therapists and psychotherapy traditions in respect to the value they place on informed consent (Somberg et al., 1993; Croarkin et al., 2003; Goddard et al., 2008). These studies reveal that psychiatrists and practitioners of insight-oriented therapies are more skeptical about the feasibility and value of informed consent (e.g., Goddard et al., 2008). To date, there has been scant investigation of therapists' views about the disclosure of process research to clients, including whether providing such information is of value to clients. Against these uncertainties, it has been proposed that the importance of informed consent is likely to be underestimated by many therapists, perhaps on the grounds that disclosure and consent are necessarily procedural in nature and cannot be obtained via a one-time disclosure (Johnson-Greene, 2007). In response to this, healthcare ethicists have argued that even if aspects of understanding how psychotherapy works necessitate procedural and ongoing consent, adequate verbal, and written information should also be provided to prospective clients at the outset (Blease et al., 2018).

### Standard Web-Based Information on CBT

Web-based information provided by leading health authorities in the US and UK provides one source of information about what is disclosed to clients about psychotherapy. For example, web-based resources provided by the NIMH explain that “[In CBT] [t]he therapist helps the client to learn how to identify distorted or unhelpful thinking patterns, recognize and change inaccurate beliefs, relate to others in more positive ways, and change behaviors accordingly” (NIMH, 2014). In the UK, the NHS's ‘Health A-Z' website states that “You and your therapist will analyse your thoughts, feelings and behaviors to work out if they're unrealistic or unhelpful and to determine the effect they have on each other and on you. Your therapist will be able to help you work out how to change unhelpful thoughts and behaviors” (2016). The UK's (National Institute for Clinical Excellence (NICE), 2009) Guidelines and the Royal College of Psychiatrists (2018) provide similar advice. In all cases, the resources focus on the specific techniques of CBT. In the case of depression, the core theoretical view of CBT (with its origins in the work of Aaron Beck) proposes that depression is the result of cognitive distortions. On this view, the specific techniques of CBT help the client to identify and to challenge the validity of maladaptive depressive thoughts, and to adopt (what Beck refers to as) more realistic thinking patterns and behaviors (Beck, 1979).

### Common Factors and CBT

In psychotherapy research there is continued debate about the relative efficacy of specific treatment factors. The so-called “Dodo bird verdict” is the claim that different versions of psychotherapy are equally effective. In support of this theory, some researchers have recently argued that while randomized controlled trials (RCTs) in psychotherapy show that CBT is effective for depression, a broad range of psychological treatments (including behavioral, cognitive, and interpersonal therapies) are also helpful in treating depression (Cuijpers et al., 2008); this research therefore challenges the idea that specific treatment techniques are the most important factors precipitating successful outcome (Wampold and Imel, 2015). The common factors hypothesis is one theory that supports the Dodo bird verdict; this hypothesis claims that it is the factors that are common across different versions of psychotherapy which principally explain their therapeutic benefits. These factors include therapist empathy, therapist positive regard, a good working alliance between client and therapist, and positive expectations on the part of both the client and the therapist.

Such common factors do not happen in a vacuum: they depend on the employment of the specific treatment techniques associated with the particular form of psychotherapy being practiced. In other words, a presumption associated with common factors is the use of some form of credible, treatment technique—as opposed to any *particular* specific treatment techniques *per se*. This is because common factors (such as working alliance, and therapist and client expectations) are dependent on the scaffolding of a psychotherapy theory replete with a rationale and techniques (Wampold and Imel, 2015).

In light of the limitations of RCTs in yielding evidence of the causal efficacy of specific factors, process studies in psychotherapy research probe the correlation between components of psychotherapy and treatment outcome, and robust findings from this research domain show that the “common factors” appear to account for a significantly higher percentage of variability in outcomes compared to specific CBT techniques (Lambert and Barley, 2001; Huppert et al., 2006; Crits-Christoph et al., 2011; Wampold et al., 2017). However, process studies should also be interpreted cautiously since correlation does not necessarily imply causation. Moreover, the Dodo bird verdict and the common factors hypothesis remain somewhat controversial among psychotherapy researchers (Beutler, 2002; Marcus et al., 2014; Cuijpers, 2016).

In summary, the common factors are associated with therapeutic outcome even if—similar to specific factors—there is no direct evidence that these factors causally mediate change. It is worth highlighting that among leading proponents of CBT both the treatment's highly specific techniques *and* common factors are asserted to be causally relevant to the therapeutic process (Beck, 1979, 2011).

### The Medical Model vs. the Contextual Model

It is worth drawing attention to a distinction in psychotherapy literature proposed by Wampold (Wampold, 2015; Wampold and Imel, 2015). Wampold characterizes the debate about evidence in psychotherapy as a clash between two kinds of explanatory model. The dominant explanatory model in psychotherapy practice—which he dubs the “Medical Model”—interprets the specific treatment techniques of psychotherapy as critical to the success of treatment. On this perspective, certain psychotherapy modalities are expected to be superior to others, and the role of the common factors, including therapist-client relationship, are thought to be much less important to treatment outcome (Wampold and Imel, 2015). Wampold differentiates this from what he describes as the “Contextual Model” of psychotherapy: this framework embraces and builds on the common factors hypothesis to explain outcomes research. Wampold argues that findings (such as the Dodo bird verdict) are explained by a constellation of fundamental, interrelated ingredients in therapy; ingredients include: “treatments with a cogent rationale that is accepted by the client, administered by a therapist who believes in the treatment and who the client believes understands the client and has the expertise to help, and contain therapeutic actions that lead to some health-promoting change will be effective” (Wampold and Imel, 2015, p. 79). Importantly, according to the Contextual Model, the context behind the particular implementation of specific factors, including the quality of the client-therapist relationship, is considered to have the most impact on outcome. Decisions about which model best fits the evidence have implications for informed consent processes, including what is ethically disclosed to individuals (Blease et al., 2016c; Gaab et al., 2016).

### Objectives of the Study

Web-based information about CBT provided by leading health authorities does not explicitly refer to the common factors in psychotherapy. By ignoring the importance of common factors, such as those related to the client-therapist relationship, this information appears consistent with the Medical Model of psychotherapy. We suggest, in light of psychotherapy research, that the question of whether common factors might usefully be included in disclosure information is pertinent in helping to address the ethical debate about what should be communicated to clients (Blease, 2015; Blease et al., 2018); hence our research question: Does information disclosure about common factors affect lay opinions about the relative importance of common and specific factors in treatment outcome? We aimed to discover whether standard information disclosures about CBT might usefully be expanded to reflect both professional attitudes and evidence from psychotherapy research, on the relevance of these factors as either mediators of change, or (at the least) significantly correlated with successful treatment outcome.

Specifically, our goal was to investigate opinions about how CBT works when laypersons are given ecologically valid information disclosures encompassing descriptions not just of the specific factors of CBT but also of the common factors. We use the term “opinions” because we assume that the lay public does not have fixed or stable attitudes about CBT. We conducted an experiment by randomizing lay participants to one of three different disclosure manipulations: No Disclosure, Standard CBT Disclosure, and Common Factors Disclosure (the latter, as emphasized above, also encompassing some information about the specific factors of CBT).

Our aim was to assess how participants ranked the therapeutic importance of various common factors in comparison to the specific techniques of CBT, and to test whether disclosures influenced opinions about the effectiveness of CBT. We predicted that participants provided with Standard CBT Disclosure would rate the specific techniques of psychotherapy more highly than participants allocated to the Common Factors disclosure. We also predicted that participants assigned to the Common Factors Disclosure would rate the common factors of psychotherapy more highly than those assigned to the Standard CBT Disclosure.

While the extent of information disclosure remains unclear across different psychotherapy modalities, it is conceivable that some referring physicians and therapists do not disclose detailed information about the nature of treatment during informed consent processes. Therefore, we also decided to include a No Disclosure condition. Our aim was to assess what difference, if any, this made to participants' opinions about how CBT works. A further reason for including this condition was to investigate the adequacy of possible justifications for omissions in disclosures. For example, some therapists may consider disclosure of both specific and common factors to be redundant on the grounds that prospective clients will intuitively grasp these factors as relevant to the success of treatment. We therefore included a No Disclosure condition to explore the rationale for any such claims: our aim was to investigate how participants' responses would differ from their response to the CBT and Common Factors Disclosures.

In light of limited, as well as mixed, findings about the effects of the gender of clients and psychotherapists on treatment outcome, we were also curious whether participants' gender would make a difference in opinions about how CBT works, and whether participants would perceive gender to be an influential factor in clients' opinions about the treatment (Parker et al., 2011; Staczan et al., 2017). Against these inconsistent and therefore inconclusive findings on gender and psychotherapy, we had no clear prediction about whether gender would make a difference to participants' responses. Finally, we also investigated whether prior knowledge of CBT influenced opinions about how CBT works.

We know of no other studies that have aimed to examine whether the content of information provided in psychotherapy (or its omission) influences lay views about psychological treatments.

## Methods

### Participants

We used the crowdsourcing tool Amazon Mechanical Turk (www.mturk.com) to recruit participants for an online study lasting an average of 8.25 min and paying $3.64 per hour on average. Inclusion criteria were that the participants had to be U.S. citizens, aged 18 years or older, and they could not have been treated previously with psychotherapy. All study procedures were approved by the Ethics Research Committee at University College Dublin, and all participants provided online informed consent.

### Procedure and Measures

All study procedures were carried out using the Qualtrics online survey platform (www.qualtrics.com). Participants were first asked a series of demographic questions, including age, gender, race, education, and employment status. They were then randomized to one of six scenarios that were constructed according to a 2 × 3 factorial design, which crossed the sex of the client in the scenario (male vs. female) with the type of consent provided (explanation of the specific factors in *CBT* vs. explanation of the *Common Factors* across psychotherapies vs. *No Disclosure* at all). The scenarios for the three types of consent with a female client are shown in Appendix A in Supplementary Material. Aside from client gender, the three scenarios with male clients were identical. On practical grounds we restricted the description in the Common Factors disclosure to those factors with the greatest reported effect size across aggregated meta-analyses (these were: working alliance, empathy, and positive regard) (Greenberg et al., 2001; Horvath et al., 2011; Wampold and Imel, 2015). The Common Factors Disclosure also included a brief description of the specific factors in CBT to preserve the ecological validity of the disclosure (shown in Appendix A in Supplementary Material).

To assess participants' opinions regarding the relative importance of various components of psychotherapy we created five statements about factors that might be important to the success of psychotherapy. In light of meta-analyses of process research, it would also have been possible to expand the number of common factors in the study (Wampold and Imel, 2015). For example, we might also have chosen to include therapist congruence/genuineness, and to differentiate goal consensus and patient-therapist alliance. However, we decided to restrict the study to four common factors statements: three of which were mentioned in the Common Factors Disclosure, and one of which was not. As shown in Appendix B in Supplementary Material, one statement describes some of the specific components of CBT, and four of the statements reflect common factors (empathy, working alliance, and positive regard). In addition, we included in our list of statements Positive Expectations about treatment as an additional common factor in order to gauge whether patients might perceive expectancy (or placebo effects) as a relatively effective component in successful treatment outcome (Blease et al., 2016b; Trachsel and Gaab, 2016).

After reading the scenario, participants were presented with a series of 10 questions that asked them to choose which of two statements they considered the more important in treating the client. The 10 pairs of statements represented all possible combinations of the five statements, and both the order of the questions, and the order of the two answer choices were randomized. We then used Thurstone's method of paired comparisons to determine for each participant a rank ordering of their preferences for the five statements (Thurstone, 1927). This method produces for each statement a value that ranges from 0 to 4, where zero means that the statement was rated as more important than none of other four statements, and four means that the statement was rated as more important that all four other statements.

Next, participants were asked to arrange the five statements in order from most to least important for treatment of the client. This method also produced a value ranging from 0 to 4, where 0 means that the statement was ranked least important, and 4 means the statement was ranked most important. By asking participants to produce a rank ordering of the five statements in two distinctly different ways, we were able to assess the degree to which they produced consistent rank orderings, which we considered a proxy for attention to the task. We chose to exclude participants for whom the Spearman correlation between these two measures was < 0.30.

The methods used were evaluative and not merely a test of participants' memory and attention to the task since, while the disclosure statements varied in content, no statement informed respondents about which factors to consider as most important. As noted, the No Disclosure condition included a statement about patient expectations; and while the Standard CBT Disclosure only provided information about specific factors and not common factors, we assumed that participants would also have intuitive opinions about the value of various patient and therapist factors in psychotherapy that could not be determined a priori.

Finally, if the correlations between the two rank ordering methods were sufficiently high (i.e., >0.50), we planned to average across the two measures to produce a single index of participants' beliefs regarding the relative importance of the five components of psychotherapy described in the statements.

## Results

### Participants

Initially, 805 persons consented to participate. However, 4 dropped out immediately after providing consent, 110 were excluded because they had previously been in psychotherapy, 7 were excluded because they were not U.S. citizens, and 79 were excluded because they did not provide responses for any of the dependent variables. This left 605 participants who met inclusion criteria and completed the study. To check that they were paying attention during the study, for each participant we computed the Spearman correlation between the two methods that were used to rank order participant preferences for the five factors in psychotherapy. This correlation indexes the degree to which each participant produced a consistent ordering of the factors. We excluded the 98 participants who had a correlation below 0.30, presuming that such low correlations suggested a substantial lack of attention to the task. After these exclusions, the final sample size for all reported analyses was 507.

The sample was 55% female, 80% Caucasian, and the mean age was 38 (SD = 13, range 18–76). The sample was well-educated—all participants had at least a high school diploma or GED, and 63% had graduated from college. Seventy-four percent of the sample was employed. Eleven percent of the sample had been diagnosed with depression at some point in their lives. Since we deliberately chose participants who had never had any form of psychotherapy, prior knowledge of CBT was low—only 28% had ever heard of CBT.

### Reliability and Descriptive Statistics for the Rank Ordering Measures

The Spearman correlations between the two methods for assessing participants' rank ordering of the five factors ranged from 0.60 to 0.84. Given these relatively high correlations, we averaged across the two measures to produce a single composite measure of preference for each factor. Reliabilities (Cronbach's alpha) for these measures were good, and ranged from 0.77 to 0.92. See Table 1 for descriptive statistics on the five composite measures of participants' ratings of the relative importance of the five factors to treatment outcome.

**Table 1:** Descriptive statistics for main outcome measures.

**Component of therapy**	**Reliability**	**Mean**	**SD**
Empathy	0.84	1.98	1.15
Specific factors of CBT	0.87	2.30	1.32
Working alliance	0.77	2.58	1.02
Expectations	0.92	1.42	1.38
Positive regard	0.90	1.73	1.34

*Reliability was assessed with Cronbach's alpha*.

### Primary Tests of Hypotheses

For the main analyses, we performed 2 × 3 factorial analyses of variance for each of the five dependent variables, with client gender and disclosure type as the independent variables. Since we conducted five separate tests, we used a Bonferroni correction to protect against inflation of Type I error. Thus, we considered significant only those *p*-values that were below.01. The interaction terms were all non-significant (*p* > 0.50 for all tests). The main effect for client sex was significant only for the Specific variable, *F*_(1, 501)_ = 4.35, *p* = 0.04. In particular, there was a small, but statistically significant tendency for raters to report that the Specific factor was more important when the client was male as compared to female. However, the effect size was small, accounting for only one percent of the variance (η_*p*_^2^ = 0.01).

**Figure 1:**
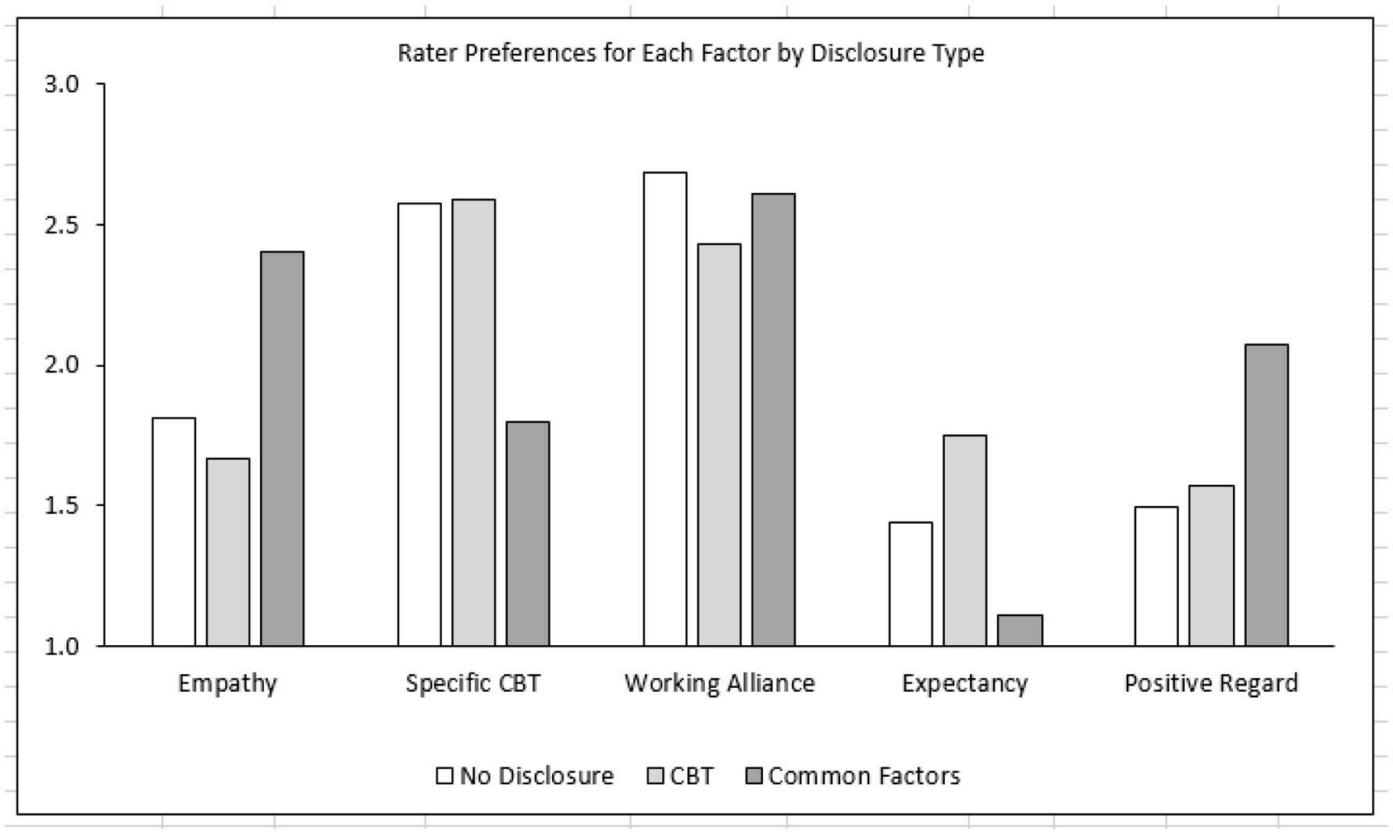
Rater preferences for each factor by disclosure type.

As shown in Table 2 and illustrated in Figure 1, the main effect for disclosure type was statistically significant for four of the five dependent variables. As predicted, Tukey *post-hoc* tests revealed that the Common Factors and CBT disclosure conditions differed from each other in systematic ways. As compared to the CBT condition, participants in the Common Factors condition rated the Empathy and Positive Regard factors as significantly more important to treatment outcome (*p* < 0.001, *d* = 0.66; and *p* = 0.001, *d* = 0.38, respectively), and they rated the Specific components of CBT as significantly less important to outcome (*p* < 0.001, *d* = 0.62). Expectancy was rated very low for both conditions, but the Expectancy ratings for the Common Factors condition were significantly lower than the CBT condition (*p* < 0.001, *d* = 0.47). The Working Alliance was rated high in both conditions, with no significant difference between the Common Factors and CBT conditions.

**Table 2:** Tests of main effects of disclosure condition.

**Factor**	**Statistical test**	**Effect size (η*_***p***_*^**2**^)**
Empathy	*F*_(2, 501)_ = 21.71, *p* < 0.001	0.08
Specific factors of CBT	*F*_(2, 501)_ = 22.85, *p* < 0.001	0.08
Working alliance	*F*_(2, 501)_ = 2.66, *p* = 0.07	0.01
Expectancy	*F*_(2, 501)_ = 9.25, *p* < 0.001	0.04
Positive regard	*F*_(2, 501)_ = 10.21, *p* < 0.001	0.04

Interestingly, there were no significant differences between the No Disclosure and the CBT conditions. In the absence of any disclosure, participants rated the five factors in a manner that was very similar to the CBT disclosure. In particular, they rated the Working Alliance and CBT factors as more important to treatment outcome, and they rated Empathy, Expectancy, and Positive Regard as less important to outcome.

The majority of participants in all three conditions agreed that they would undergo CBT treatment if it were recommended to them by their doctor (89% of participants allocated to CBT disclosure; 82% in Common Factors, and 78% in the No Disclosure group). However, those in the CBT disclosure were significantly more likely to agree to undergo CBT than those allocated to the Common Factors [$\chi_{\left(1\right)}^{2}$ = 3.88, *p* = 0.049] or No Disclosure [$\chi_{\left(1\right)}^{2}$ = 8.76, *p* = 0.003] conditions.

A little more than a quarter of the participants (28.4%) had previously heard of CBT. To determine whether prior knowledge of CBT affected the study results, we added a covariate that indicated whether the participant had prior knowledge of CBT. This covariate had no significant effect on our analyses of four of the five dependent variables. There was a significant effect for the Specific Factors variable (*p* = 0.04), but the effect size was small—partial eta-squared was 0.008, indicating that a little < 1% of the variance in participants' ratings of the Specific Factors variable could be accounted for by whether or not they had prior knowledge of CBT.

We unpacked this effect by conducting an independent samples *t*-test within each of the three disclosure conditions in which we compared the ratings of the Specific Factor by participants who had previous knowledge of CBT against the ratings of those who did not have prior knowledge of CBT. The result was significant in the No Disclosure condition (2.92 vs. 2.35, respectively, *p* = 0.003), but not significant in the Standard CBT or Common Factors conditions. These results indicate that prior knowledge of CBT yielded somewhat higher ratings of the Specific Factors, but *only* in the No Disclosure condition.

## Discussion

### Summary of Main Findings

As predicted, the Common Factors disclosure (as compared to the Specific Factors disclosure) increased participants' ratings of the relative importance of Empathy and Positive Regard to treatment outcome, and decreased ratings of the Specific Factors of CBT. Although Expectancy was rated very low in both conditions, participants in the Common Factors condition rated Expectancy as significantly less important to outcome, than the participants in the Standard CBT condition. The Working Alliance was rated highly in both conditions, with no significant differences between the Common Factors and the Standard CBT disclosures.

These results can be interpreted in the light of the content of the Common Factors and Standard CBT disclosures. The Common Factors disclosure specifically references Empathy and Positive Regard as important to treatment outcome; and as compared to the Standard CBT disclosure, it places less emphasis on the Specific Factors of CBT. Neither disclosure mentions Positive Expectations and the ratings for this factor were correspondingly low in both conditions, albeit lower for the Common Factors condition than the Standard CBT condition. Despite the fact that the Working Alliance factor was specifically mentioned in the Common Factors disclosure and not mentioned in the Standard CBT disclosure, it was rated highly in both conditions. This factor was also highly rated in the No Disclosure condition, which suggests that laypersons intuitively consider a good working alliance as important, and that a ceiling effect may have been present.

We had no specific hypotheses about how the No Disclosure condition would affect participants' ratings, but instead, we included the condition as a control. It appears that in the absence of any disclosure, laypersons rate the five factors in a very similar manner to the Standard CBT disclosure. In particular, they rate the Working Alliance and the Specific Factors of CBT as more important to treatment outcome, and rate Expectations, Empathy, and Positive Regard as less important. However, given research indicating that these three common factors are correlated with treatment outcome (Lambert and Barley, 2001; Crits-Christoph et al., 2011; Wampold and Imel, 2015), this finding suggests that disclosing the importance of these common factors may be an important component of the informed consent process.

The common factors of psychotherapy can be viewed as the “softer” components of treatment since they relate to the qualities of the interpersonal relationship between client and clinician. In contrast, the specific factors can be thought of as the “harder” components of treatment in that they involve the technical aspects of treatment. Our results suggest that laypersons may intuitively consider that the interpersonal relationship is of less importance for treatment outcome in CBT than the proficiency of the clinician in carrying out the technical aspects of psychotherapy. However, this interpretation might be criticized because the participants in the No Disclosure condition rated the Working Alliance factor as important, and the working alliance is considered to be one of the common factors. We would counter this by noting that the working alliance concept is the least relational of all the common factors in that it focuses attention on the *professional* aspects of the relationship, as exemplified by its focus on agreement between client and clinician on the goals and tasks of psychotherapy, and by inclusion of the modifier “working” in the construct's name.

These findings for psychotherapy are reminiscent of patient preferences in regard to surgeons. In the trade-off between technical expertise and bedside manner, most patients report that they can accept that a surgeon who has poor interpersonal skills, so long as he or she has excellent surgical skills (Fung et al., 2005). Such trade-offs are unfortunate in that a poor interpersonal relationship may impede communication between patient and physician, potentially resulting in misdiagnoses when the physician fails to elicit important, relevant information, and poor compliance with treatment recommendations when the physician fails to adequately communicate the treatment plan to the client (Hojat et al., 2011; Derksen et al., 2013; Kelley et al., 2014). Of course, the problems associated with a relatively poor client-clinician relationship are magnified in the case of psychotherapy where the treatment itself is delivered through the interpersonal relationship.

While the majority of participants agreed that they would undergo CBT if it was recommended to them, significantly more participants allocated to a Standard CBT disclosure were willing to undergo the treatment than those in the Common Factors and No Disclosure conditions. This may not be surprising given that the Standard CBT disclosure emphasized the specific treatment techniques of CBT; moreover, while the Common Factors disclosure did include a brief description of specific CBT techniques, these were not conveyed in the same detail as the Standard CBT disclosure. In future studies (including both empirical and ethical research), it will be important to investigate how psychological treatments are conceptualized or labeled, and the consequences of such descriptive labeling as well as information disclosures, in influencing patients' willingness to undergo such treatments.

Finally, only 28% of participants had previously heard of CBT. This was surprising given that CBT is the most widely used form of psychotherapy worldwide. Individuals who had previously heard of CBT rated the specific factors slightly higher—but only in the No Disclosure condition. When no information was forthcoming, prior knowledge likely primed participants to rank specific factors higher than the other listed factors; curiously, however, this foreknowledge did not appear to bias participants' opinions in the Standard CBT disclosure. One possible explanation for this finding is that those who had previously heard of CBT may have already been predisposed to rate the specific factors as more important than the common factors, and the CBT disclosure may not have been able boost that bias any further.

Interestingly, our findings were not modified by the gender of the clients in the scenarios. Against this experimental finding, it should be pointed out that there is some evidence that gender may implicitly influence level of perceived therapist empathy in clinical practice, with some evidence suggesting female practitioners may be more effective at displaying empathic behavior (Gleichgerrcht and Decety, 2013; Howick et al., 2017).

### Strengths and Limitations

Ethical and practical constraints limit investigation of what is disclosed to clients during psychotherapy sessions, and how this information influences clients' attitudes about treatment, as well as treatment outcomes. Our study therefore focused on the opinions of psychotherapy-naive individuals about the effective components of CBT in light of various disclosure conditions. Our aim was restricted to examining how initial disclosures might influence prospective clients' attitudes about CBT in light of widely accessible information—what we described as “standard disclosures,” such as that found on the NHS Choices website—information which may be read or recommended by doctors prior to individuals commencing psychotherapy (NHS, 2016). In this way, our investigation yields some insight about how the content of typical CBT information, and non-disclosure of any information, may influence psychotherapy-naive participants' opinions about how this psychological treatment works.

One potential criticism of this study is that it should come as no surprise that individuals presented with vignettes about common factors in CBT also report these as more important, and vice versa for specific factors. There are two responses to this. First, we are happy to bite the bullet: the aim of the study is expressly to investigate informed consent, and we hoped that the information contained in the vignettes would make a difference to lay opinions about how therapy works. Of course, it is also important to note that in clinical contexts retention of information may be even lower for patients as compared to individuals participating in an experiment. Notwithstanding these limitations, our study indicates that for individuals who are naïve to psychotherapy, providing no information disclosure has similar consequences as providing a standard CBT disclosure. Second, as mentioned previously, the disclosure statements were still open to interpretation among participants: it was left open to individuals to evaluate the importance of the various common and specific factors since no hierarchical ordering was provided in any disclosure. The results clearly showed that omission or inclusion of information in disclosures influenced participants' opinions about how CBT works.

By using random allocation of participants to different disclosure scenarios, we were able to compare the effects of the information provided on participants' opinions about specific and common factors in CBT. A particular strength of the study was the use of both Thurstone's method of paired comparisons and the rank ordering of statements about specific and common factors, which allowed us to assess the degree to which participants made similar judgments across different rating tasks—a proxy for attention to the task. We then excluded participants who were inconsistent in their ratings and were presumably inattentive or confused by the task. Another important strength was that the majority of participants (84%) were consistent across both methods in how they ranked the factors provided. However, one weakness of these methods of assessment was that participants were not allowed to rate two factors as equally important to treatment outcome. One strategy to get around this potential problem would have been to request that participants separately rate each factor on using a scale measure. Given that these participants were naïve to psychotherapy, we were concerned that many participants might be tempted to provide the same ratings for all factors as a default answer when confronted with a difficult choice. Indeed, findings suggests that, when offered neutral options, research participants may invest less effort in their responses (Krosnick et al., 2002). Thus, the methods we chose have the practical advantage of forcing participants to make decisions about the relative effectiveness of the five factors.

A further limitation was the uneven representation of the “common” (*n* = 4) vs. “specific” (*n* = 1) factors. Indeed, had participants responded randomly, there would be an 80% chance that one of the common factors would be the “most important” and only a 20% chance that the specific factor would be ranked “most important.” One possible response might have been to provide a list of alternative yet synonymous descriptors of specific factors to provide an even number of factors. While we acknowledge this shortcoming, it is noteworthy that the specific factor was ranked highest in the CBT Disclosure condition and second highest in the No Disclosure condition.

Another potential criticism is the disclosure scenarios (Appendix A in Supplementary Material) and statements about common and specific factors (Appendix B in Supplementary Material) which purport to describe “how CBT works.” It might be argued that there is not sufficient evidence to support the claim that common or specific factors accurately describe *how* CBT works—that RCTs and process research in psychotherapy have not identified the causal factors responsible for change in CBT, and the most we can claim is that specific techniques of CBT and common factors are correlated with treatment outcome. In response we would counter that standard disclosures to CBT are equally unambiguous in advising prospective clients that the various techniques in CBT are causal mechanisms which mediate therapeutic change; therefore, this criticism might also be leveled at standard information about CBT (Blease, 2015). While we concede that more truthful disclosure statements might have included more elaborate and accurate qualifications about CBT, we decided that overcomplicating the disclosures may have had the consequence of confusing participants by overloading them with information.

Future studies might investigate whether clients' opinions about the effective factors in CBT are likely to change as a result of procedural knowledge gained through participation in the process of CBT, and indeed, whether a one-off disclosure is sufficient to establish changes in clients' knowledge about how therapy works. Future research might be aimed at determining if and when information disclosures lead to more enduring attitude changes in opinions about treatments, including whether revisiting disclosures about therapy is necessary to provide meaningful, ongoing client consent. Follow up studies might also usefully investigate whether disclosures influence individuals' decisions on whether to embark on psychological treatment, on what factors to consider when choosing a therapist, and whether the type of disclosure influences clients' level of trust in their therapists.

## Conclusions and Recommendations

CBT is a form of psychotherapy in widespread use in the UK, the US, and worldwide; it has the potential to benefit patients suffering from a range of mental disorders and conditions, including depression. When disclosures emphasize a “Medical Model” of CBT by focusing exclusively on the specific techniques of the treatment, our study suggests that prospective clients may undervalue the interpersonal expertise of therapists (in particular, empathy, and positive regard). Interestingly, No Disclosure was similar to the Standard CBT Disclosure: in both cases, clients infer that the working alliance and the specific factors of CBT are most important to outcome.

These findings have ethical implications for informed consent processes and may influence individuals' initial decisions about whether to embark on psychotherapy, or how to select therapists (Gaab et al., 2016; Blease et al., 2018). For example, it may be that when clients place a premium on the specific techniques of CBT and undervalue the common factors, this may affect their perception of successful progress within psychotherapy. It has also been argued that some clients who discontinue psychotherapy may come away with a potentially false impression that “CBT is not for me” rather than the plausible alternative perception that (for example) other factors such as those associated with therapist empathy and demeanor may have influenced their outcome (Blease, 2015). It is conceivable that adequate information about both specific and common factors in psychotherapy may help foster a trusting relationship by demystifying psychotherapy for clients, and by increasing a sense of autonomy and personal responsibility for the process (Beahrs and Gutheil, 2001; Fisher and Oransky, 2008; Trachsel et al., 2015). A recent meta-analysis has concluded that a higher level of trust in medical professionals correlates with enhanced health outcomes (Birkhäuer et al., 2017).

Our study suggests that Common Factors disclosures may elicit more accurate, evidence-based opinions about how CBT works than standard—or indeed, no—information provided about CBT. This finding is important, since it challenges the suggestion that disclosure about common factors is unnecessary and therefore unwarranted. While it is unclear whether such a stance is widespread among therapists, conceivably a laissez-faire approach to common factors disclosure might be prevalent, and defended on three grounds. First, some therapists might consider disclosure of these factors redundant on the basis that prospective clients intuitively view these as relevant to the success of therapy. Our findings suggest, however, that this cannot be taken as a given. Second, practitioners may consider disclosure of the information about therapist or client factors as tricky or awkward, and perhaps as posing a risk to the quality of the client-practitioner relationship during the first encounter. While our research does not address whether disclosure about common factors augments or diminishes the therapeutic alliance, as noted, there are reasons to believe that transparency and honesty may, in fact, build trust, possibly facilitating improved outcomes (e.g., Birkhäuer et al., 2017). Third, some may consider disclosure of common factors inappropriate on the grounds that it is the job of a good therapist to discern whether (for example) the therapeutic alliance is on track. We argue that such a standpoint neglects the duty of therapists to respect the autonomy of potential clients by providing a more comprehensive, evidence-based disclosure (Blease, 2015; Gaab et al., 2016; Blease et al., 2018).

Although our findings are exploratory and confined to an experimental set-up, they hint at valuable clinical recommendations which might easily be incorporated into routine practice. To begin, referring physicians and psychotherapists might usefully emphasize common factors (as well as specific treatment techniques) within information disclosures. Descriptions of common factors might be given in brief, accessible language, in ways that do not cause the client or the therapist to experience undue discomfort during initial therapy sessions. An example of such a disclosure might be:

“During these sessions it is important that you feel comfortable talking to me. You should also feel supported and understood, and feel like you can readily get on board with the work we will do together in the CBT sessions. If for any reason you feel uncomfortable about the progress we are making, it is important that we talk about that. We can try to work through these problems, but it may be that a different version of psychotherapy or a different therapist may work better for you. While I do not expect this to happen, in some cases another kind of psychotherapy or therapist might be more suitable for you and that is nobody's fault.” (Adapted from Blease et al., 2018).

An additional recommendation flows from our finding that individuals with foreknowledge of CBT tended to prioritize the value of specific factors in the No Disclosure condition. In clinical contexts, it is certainly possible that if individuals are asked whether they have heard of CBT, and answer affirmatively, that such persons are less likely to be furnished with a full informational disclosure. Our research is at least suggestive that this response would be a mistake, and that adequate disclosure must be provided to *all* prospective clients, regardless of whether their familiarity with CBT has been established.

In closing, we emphasize that future studies should investigate whether these findings in this study translate to clients, including if disclosure statements, and disclosure processes affect clients' willingness to undergo CBT and other psychological treatments. Larger scale research might also investigate whether disclosure practices, including the disclosure of common factors enhance or undermine client outcomes across different versions of psychotherapy. Additionally, future research might usefully investigate the possibility of augmenting placebo or positive expectancy effects among clients in an open and ethical way (Blease et al., 2016a). Finally, it is also conceivable that current disclosure practices may undermine treatment outcomes for some clients with certain conditions, and this too requires further investigation (Geraghty and Blease, 2016). Our study provides only a preliminary contribution to the overlooked question about how to improve patient resources, and disclosure processes to psychological treatments.

## Data sharing

The questionnaire and dataset are available from the corresponding authors.

## Ethics Statement

This study was approved by the Ethics Research Committee at University College Dublin.

## Author Contributions

CB conceived the study and wrote the first draft. CB and JK revised the experiment, co-authored the paper, and revised the draft several times until both signed off on the final manuscript. JK analyzed the data.

### Conflict of Interest Statement

The authors declare that the research was conducted in the absence of any commercial or financial relationships that could be construed as a potential conflict of interest.

## References

[B1] American Medical Association (2006). AMA Code of Medical Ethics. Opinion 8.08–Informed Consent. Available online at: www.ama-assn.org/ama/pub/physician-resources/medical-ethics/code-medical-ethics/opinion808.page? (Accessed December 5, 2017).

[B2] American Psychological Association (2010). 10.01 Informed Consent to Psychotherapy. Ethical Principles of Psychologists and Code of Conduct. Washington, DC: American Psychological Association.

[B3] Beahrs J. O. Gutheil T. G. (2001). Informed consent in psychotherapy. Am. J. Psychiatry, 158, 4–10. 10.1176/appi.ajp.158.1.4 11136625

[B4] Beck A. T. (1979). Cognitive Therapy and the Emotional Disorders. London, UK: Penguin.

[B5] Beck J. S. (2011). Cognitive Behavior Therapy: Basics and Beyond. New York, NY: Guilford Press.

[B6] Beutler L. E. (2002). The dodo bird is extinct. Clin. Psychol. Sci. Pract. 9, 30–34. 10.1093/clipsy.9.1.30

[B7] Birkhäuer J. Gaab J. Kossowsky J. Hasler S. Krummenacher P. Werner C. Gerger H. (2017). Trust in the health care professional and health outcome: a meta-analysis. PLoS ONE 12:e0170988. 10.1371/journal.pone.0170988 28170443 PMC5295692

[B8] Blease C. Colloca L. Kaptchuk T. J. (2016a). Are open-Label Placebos Ethical? Informed consent and ethical equivocations. Bioethics 30, 407–414. 10.1111/bioe.12245 26840547 PMC4893896

[B9] Blease C. Kelley J. M. Trachsel M. (2018). Informed consent in psychotherapy: implications of evidence-based practice. J. Contemp. Psychother. 48, 69–78. 10.1007/s10879-017-9372-9

[B10] Blease C. Trachsel M. Holtforth M. G. (2016b). Paternalism and placebos: the challenge of ethical disclosure in psychotherapy. Verhaltenstherapie 26, 22–30. 10.1159/000442928

[B11] Blease C. R. (2015). Talking more about talking cures: cognitive behavioural therapy and informed consent. J. Med. Ethics Medethics. 41, 750–755. 10.1136/medethics-2014-102641 25887514

[B12] Blease C. R. Lilienfeld S. O. Kelley J. M. (2016c). Evidence-based practice and psychological treatments: the imperatives of informed consent. Front. Psychol. 7:1170. 10.3389/fpsyg.2016.01170 27559322 PMC4979245

[B13] British Association for Counseling Psychotherapy (2016). ‘Ethics for counseling and psychotherapy', Ethical Framework for Good Practice in Counseling and Psychotherapy. Available online at: http://www.bacp.co.uk/ethical_framework/new_ef.php (Accessed July 6, 2017).

[B14] Clark D. M. (2011). Implementing NICE guidelines for the psychological treatment of depression and anxiety disorders: the IAPT experience. Int. Rev. Psychiatry 23, 318–327. 10.3109/09540261.2011.606803 22026487 PMC3212920

[B15] Crits-Christoph P. Gibbons M. B. Hamilton J. Ring-Kurtz S. Gallop R. (2011). The dependability of alliance assessments: the alliance-outcome correlation is larger than you might think. J. Consult. Clin. Psychol. 79, 267–278. 10.1037/a0023668 21639607 PMC3111943

[B16] Croarkin P. Berg J. Spira J. (2003). Informed consent for psychotherapy: a look at therapists' understanding, opinions, and practices. Am. J. Psychother. 57:384. 10.1176/appi.psychotherapy.2003.57.3.384 12961822

[B17] Cuijpers P. (2016). Are all psychotherapies equally effective in the treatment of adult depression? The lack of statistical power of comparative outcome studies. Evid. Based Ment. Health 19, 39–42. 26984413 10.1136/eb-2016-102341 PMC10699414

[B18] Cuijpers P. van Straten A. Andersson G. van Oppen P. (2008). Psychotherapy for depression in adults: a meta-analysis of comparative outcome studies. J. Consult. Clin. Psychol. 76:909. 10.1037/a0013075 19045960

[B19] Derksen F. Bensing J. Lagro-Janssen A. (2013). Effectiveness of empathy in general practice: a systematic review. Br. J. Gen. Pract. 63, e76–e84. 10.3399/bjgp13X660814 23336477 PMC3529296

[B20] Fisher C. B. Oransky M. (2008). Informed consent to psychotherapy: Protecting the dignity and respecting the autonomy of patients. J. Clin. Psychol. 64, 576–588. 10.1002/jclp.20472 18381749

[B21] Fonagy P. Rost F. Carlyle J. A. McPherson S. Thomas R. Pasco Fearon R. M. Taylor D. (2015). Pragmatic randomized controlled trial of long-term psychoanalytic psychotherapy for treatment-resistant depression: the Tavistock Adult Depression Study (TADS). World Psychiatry 14, 312–321. 10.1002/wps.20267 26407787 PMC4592654

[B22] Fung C. H. Elliott M. N. Hays R. D. Kahn K. L. Kanouse D. E. McGlynn E. A. Shekelle P. G. (2005). Patients' preferences for technical versus interpersonal quality when selecting a primary care physician. Health Serv. Res. 40, 957–977. 10.1111/j.1475-6773.2005.00395.x 16033487 PMC1361181

[B23] Gaab J. Blease C. Locher C. Gerger H. (2016). Go open: A plea for transparency in psychotherapy. Psychol. Conscious. Theor. Res. Prac. 3:175 10.1037/cns0000063

[B24] General Medical Council (2010). Good Medical Practice. Available online at: www.gmc-uk.org/Good_Medical_Practice_Archived.pdf_51772200.pdf (Accessed December 5, 2017).

[B25] Geraghty K. J. Blease C. (2016). Cognitive behavioural therapy in the treatment of chronic fatigue syndrome: a narrative review on efficacy and informed consent. J. Health Psychol. 23, 127–138. 10.1177/1359105316667798 27634687

[B26] Gleichgerrcht E. Decety J. (2013). Empathy in clinical practice: how individual dispositions, gender, and experience moderate empathic concern, burnout, and emotional distress in physicians. PLoS ONE 8:e61526. 10.1371/journal.pone.0061526 23620760 PMC3631218

[B27] Goddard A. Murray C. D. Simpson J. (2008). Informed consent and psychotherapy: an interpretative phenomenological analysis of therapists' views. Psychol. Psychother. Theor. Res. Prac. 81, 177–191. 10.1348/147608307X266587 18086340

[B28] Greenberg L. S. Watson J. C. Elliot R. Bohart A. C. (2001). Empathy. Psychother. Theor. Res. Pract. Train. 38:380 10.1037/0033-3204.38.4.380

[B29] Hojat M. Louis D. Z. Markham F. W. Wender R. Rabinowitz C. Gonnella J. S. (2011). Physicians' empathy and clinical outcomes for diabetic patients. Acad. Med. 86, 359–364. 10.1097/ACM.0b013e3182086fe1 21248604

[B30] Hollon S. D. Stewart M. O. Strunk D. (2006). Enduring effects for cognitive behavior therapy in the treatment of depression and anxiety. Annu. Rev. Psychol. 57, 285–315. 10.1146/annurev.psych.57.102904.190044 16318597

[B31] Horvath A. O. Del Re A. C. Flückiger C. Symonds D. (2011). Alliance in individual psychotherapy. Psychotherapy 48:9. 10.1037/a0022186 21401269

[B32] Howick J. Steinkopf L. Ulyte A. Roberts N. Meissner K. (2017). How empathic is your healthcare practitioner? A systematic review and meta-analysis of patient surveys. BMC Med. Educ. 17:136. 10.1186/s12909-017-0967-3 28823250 PMC5563892

[B33] Huppert J. D. Fabbro A. Barlow D. H. (2006). Evidence-based practice and psychological treatments. Evidence-based psychotherapy: Where practice and research meet. 131–152. 10.1037/11423-006

[B34] Johnson-Greene D. (2007). Evolving standards for informed consent: is it time for an individualized and flexible approach? Prof. Psychol. Res. Pract. 38:179.

[B35] Kelley J. M. Kraft-Todd G. Schapira L. Kossowsky J. Riess H. (2014). The influence of the patient-clinician relationship on healthcare outcomes: a systematic review and meta-analysis of randomized controlled trials. PLoS ONE 9:e94207. 10.1371/journal.pone.0094207 24718585 PMC3981763

[B36] Krosnick J. A. Holbrook A. L. Berent M. K. Carson R. T. Michael Hanemann W. Kopp R. J. (2002). The impact of “no opinion” response options on data quality: non-attitude reduction or an invitation to satisfice? Public Opin. Q. 66, 371–403. 10.1086/341394

[B37] Lambert M. J. Barley D. E. (2001). Research summary on the therapeutic relationship and psychotherapy outcome. Psychother. Theor. Res. Pract. Train. 38, 357 10.1037/0033-3204.38.4.357

[B38] Leichsenring F. Rabung S. Leibing E. (2004). The efficacy of short-term psychodynamic psychotherapy in specificpsychiatric disorders: a meta-analysis. Arch. Gen. Psychiatry 61, 1208–1216. 10.1001/archpsyc.61.12.1208 15583112

[B39] Marcus D. K. O'Connell D. Norris A. L. Sawaqdeh A. (2014). Is the Dodo bird endangered in the 21st century? A meta-analysis of treatment comparison studies. Clin. Psychol. Rev. 34, 519–530. 10.1016/j.cpr.2014.08.001 25238455

[B40] National Institute for Clinical Excellence (NICE) (2009). Clinical Guideline 90: The Treatment and Management of Depression. Available online at: http://www.nice.org.uk/nicemedia/pdf/CG90NICEguideline.pdf (Accessed July 8, 2016).

[B41] NHS (2016). Cognitive Behavioural Therapy - How it Works. Available online at: https://www.nhs.uk/conditions/cognitive-behavioural-therapy-cbt/how-it-works/ (Accessed December 14, 2018).

[B42] NIMH (2014). Mental Health Information: Psychotherapies.28801423

[B43] Norcross J. C. Karpiak C. P. Santoro S. O. (2005). Clinical psychologists across the years: The division of clinical psychology from 1960 to 2003. J. Clin. Psychol. 61, 1467–1483. 10.1002/jclp.20135 15880436

[B44] Parker G. Blanch B. Crawford J. (2011). Does gender influence response to differing psychotherapies by those with unipolar depression? J. Affect. Disord. 130, 17–20. 10.1016/j.jad.2010.05.020 20580094

[B45] Royal College of Psychiatrists (2018). Cognitive Behavioural Therapy (CBT). Available online at: https://www.rcpsych.ac.uk/mental-health/treatments-and-wellbeing/cognitive-behavioural-therapy-(cbt) (Accessed December 14, 2018).

[B46] Smith M. L. Glass G. V. (1977). Meta-analysis of psychotherapy outcome studies. Am. Psychol. 32, 752. 10.1037/0003-066X.32.9.752 921048

[B47] Somberg D. R. Stone G. L. Claiborn C. D. (1993). Informed consent: Therapists' beliefs and practices. Profession. Psychol. Res. Pract. 24, 153. 10.1037/0735-7028.24.2.153 11659698

[B48] Staczan P. Schmuecker R. Koehler M. Berglar J. Crameri A. Von Wyl A. Tschuschke V. (2017). Effects of sex and gender in ten types of psychotherapy. Psychother. Res. 27, 74–88. 10.1080/10503307.2015.1072285 26291131

[B49] Thurstone L. L. (1927). The method of paired comparisons for social values. J. Abnormal Soc. Psychol. 21, 384–400. 10.1037/h0065439

[B50] Trachsel M. Gaab J. (2016). Disclosure of incidental constituents of psychotherapy as a moral obligation for psychiatrists and psychotherapists. J. Med. Ethics 42, 493–445. 10.1136/medethics-2015-102986 27169707

[B51] Trachsel M. Grosse Holtforth M. Biller-Andorno N. Appelbaum P. S. (2015). Informed consent for psychotherapy: still not routine. Lancet Psychiatry 2, 775–777. 10.1016/S2215-0366(15)00318-1 26360886

[B52] Wampold B. E. (2015) How important are the common factors in psychotherapy? An update. World Psychiatry 14, 270–277. 10.1002/wps.20238 26407772 PMC4592639

[B53] Wampold B. E. Flückiger C. Del Re A. C. Yulish N. E. Frost N. D. Pace B. T.. (2017). In pursuit of truth: a critical examination of meta-analyses of cognitive behavior therapy. Psychother. Res. 27, 14–32. 10.1080/10503307.2016.1249433 27884095

[B54] Wampold B. E. Imel Z. E. (2015). The Great Psychotherapy Debate: The Evidence for What Makes Psychotherapy Work. New York, NY: Routledge 10.4324/9780203582015

