# Supplementary material for: Does Disclosure About the Common Factors Affect Laypersons' Opinions About How Cognitive Behavioral Psychotherapy Works?
Source: Front Psychol. 2018 Dec 21;9:2635. doi: 10.3389/fpsyg.2018.02635 (PMC6308208; doi:10.3389/fpsyg.2018.02635)
Supplement: Supplementary file 1 [file Data_Sheet_1.docx]

**Appendix A: Disclosure Scenarios**

**Standard CBT Disclosure Scenario**

A woman who is feeling very low visits her doctor. The doctor diagnoses her with depression and recommends she undergo a form of psychotherapy called cognitive behavioral therapy (CBT). The woman is unsure what CBT is but her doctor explains how it works and provides the woman with the following information in a leaflet. The doctor asks her to read it:

“Cognitive behavioral therapy (‘CBT’) is a form of psychotherapy or talking treatment which is commonly used to treat depression. It helps you deal with your problems more effectively. CBT focuses on a person’s thoughts and beliefs and how they influence a person’s mood and actions. Negative thoughts and feelings can trap you in a vicious cycle. CBT aims to help you break the vicious cycle of unhealthy thinking patterns. The therapist helps you to identify distorted thinking patterns that may be making you depressed. Your therapist will help you to recognize inaccurate thoughts and change your thinking and behavior accordingly.”

The woman decides to undergo CBT therapy.

**Common Factors Disclosure Scenario**

A woman who is feeling very low visits her doctor. The doctor diagnoses her with depression and recommends she undergo a form of psychotherapy called cognitive behavioral therapy (CBT). The woman is unsure what CBT is but her doctor explains how it works and provides the woman with the following information in a leaflet. The doctor asks her to read it:

“Cognitive behavioral therapy (‘CBT’) is a form of psychotherapy or talking treatment which is commonly used to treat depression. The therapist helps you to identify distorted thinking patterns that may be making you depressed. However, CBT works best for those clients who can agree on the tasks and goals of therapy and form a good working relationship with their therapist. It is also more effective when the therapist is empathetic (understands the client’s perspective and feelings) and supports and encourages the client throughout their time in therapy.”

The woman decides to undergo CBT therapy.

**No Disclosure Scenario**

A woman who is feeling very low visits her doctor. The doctor diagnoses her with depression and recommends she undergo a form of psychotherapy called cognitive behavioral therapy (CBT). The woman is unsure what CBT is but decides to undergo CBT anyway.
