# Supplementary material for: Does Disclosure About the Common Factors Affect Laypersons' Opinions About How Cognitive Behavioral Psychotherapy Works?
Source: Front Psychol. 2018 Dec 21;9:2635. doi: 10.3389/fpsyg.2018.02635 (PMC6308208; doi:10.3389/fpsyg.2018.02635)
Supplement: Supplementary file 2 [file Data_Sheet_2.docx]

**Appendix B: Descriptive Statements about Common and Specific Factors**

**Common Factors – Empathy**

The therapist understands the client’s problems and feelings and can see things from the client’s perspective. The therapist communicates this understanding and shows concern for the client.

**Common Factors – Working Alliance**

The therapist and the client agree about the goals and tasks of therapy. The therapist and client collaborate and form a good working alliance in trying to achieve these goals.

**Common Factors – Positive Regard**

The therapist has a warm demeanor and is reassuring toward the client. The therapist accepts that the client is finding things difficult, and encourages and supports the client.

**Common Factors – Positive Expectations**

The client expects CBT to be an effective treatment for depression. The therapist also shares the belief that CBT is an effective form of treatment for clients who are depressed.

**Specific Factors of CBT**

The therapist applies the specific principles of CBT with the client. The therapist teaches the client how to identify, evaluate and respond to depressive thoughts and behaviors using the theory and methods of CBT.
